# Willingness of Older Adults to Travel for Medical Care

**DOI:** 10.1001/jamanetworkopen.2025.60280

**Published:** 2026-02-23

**Authors:** Jeremy Burke, Tabasa Ozawa, Ying Liu, Wei Ye, Soeren Mattke

**Affiliations:** 1 The USC Brain Health Observatory, University of Southern California, Los Angeles

## Abstract

**Importance:**

Geographic distance to medical care is often thought to create obstacles to access. However, little is known about acceptable travel times for routine medical care and how willingness to travel varies by the type of medical services and individual characteristics.

**Objective:**

To survey older adults in the US to determine the limits for acceptable travel times to primary care, specialty care, and diagnostic procedures and to investigate the factors associated with their willingness to travel.

**Design, Setting, and Participants:**

In this survey study using a discrete choice experiment, a survey was fielded to participants of a nationally representative internet panel (the Understanding America Study) between April 23 and June 8, 2025. Understanding America Study panelists aged 65 years or older were eligible and invited to participate.

**Main Outcomes and Measures:**

The primary outcomes were current and maximum acceptable travel times to primary care, specialty care, and one-time diagnostic services. Linear regression was used to explore the associations between respondent characteristics and willingness to travel.

**Results:**

Of the 3390 individuals invited, 2650 respondents were included in the study (78.2% response rate). Respondents had a mean (SD) age of 72.9 (6.0) years; 1377 (52.0%) were female, and 1366 (51.5%) had an annual household income of more than $60 000. The median willingness to travel times were 67.5 (IQR, 37.5-112.5) minutes, 127.5 (IQR, 67.5-232.5) minutes, and 112.5 (IQR, 67.5-202.5) minutes for primary care, specialty care, and one-time diagnostic appointments, respectively. The majority of respondents were willing to travel at least 1 hour to receive primary care (1597 [60.3%]), specialty care (2237 [84.4%]), or one-time diagnostic care (2185 [82.5%]). Regression results revealed that individuals who had higher incomes and greater educational attainment, racial and ethnic minority individuals, and those who typically drove themselves to appointments were willing to travel longer durations; for example, those with incomes greater than $60 000 would travel 22.6 (95% CI, 13.3-31.9) more minutes to specialty care than those with lower incomes. Respondents who lived in metropolitan areas, those who reported being in fair or poor health, and those who reported difficulty traveling to medical appointments in the past were willing to travel shorter durations; for example, those who self-reported fair or poor health were willing to travel 15.0 (3.9-26.1) fewer minutes to specialty care appoints than those with better self-reported health.

**Conclusions and Relevance:**

The findings of this survey study suggest that older US adults were willing to travel for substantial durations before delaying or forgoing routine medical care, with important differences by appointment type and individual characteristics. Future research can explore willingness to travel for other types of medical appointments or procedures or for other populations.

## Key Points

**Question:**

What duration are older adults in the US willing to travel for routine medical appointments before delaying or forgoing care?

**Findings:**

In this survey study that included 2650 older adults (aged ≥65 years) from a nationally representative internet panel, the median willingness to travel times were 68 minutes, 128 minutes, and 113 minutes for primary care, specialty care, and one-time diagnostic appointments, respectively.

**Meaning:**

These findings suggest that older adults in the US are willing to travel for substantial durations before delaying or forgoing routine medical care.

## Teaser

This survey study investigates the willingness of older adults in the US to travel for routine medical care, including current and maximum acceptable travel times by appointment type and associated individual characteristics.

## Introduction

Geographic obstacles to accessing medical care are often assumed to be drivers of health disparities and poor outcomes. A 2023 study reported that travel time for medical care in the US has increased in recent years, especially for individuals living in rural areas.^1^ Given the rise in health system consolidation,^2^ hospital closures,^3^ and physician shortages and maldistribution,^4^ travel time to medical care could continue to increase in the future and may be further exacerbated by the Medicaid spending cuts in 2026, which are feared to increase financial strain on rural hospitals.^5^

While geographic distance is only one potential obstacle to access, it is critical to understand how travel time to medical care affects health care utilization and health outcomes. One strand of research has characterized the geographic obstacles to care as travel time to the nearest facility, by assuming—rather than empirically showing—that longer travel duration means poorer access. For instance, Labban et al^6^ found that socioeconomically disadvantaged and racial and ethnic minority respondents were more likely to rely on public transportation for trips to medical appointments, and the investigators thus argued that the resulting travel burden could lead to worse outcomes. Other studies considered jurisdictional boundaries as additional constraints. In a US study investigating travel time for Alzheimer disease treatment in Georgia, Li et al^7^ found that residents of rural counties would expect to have significantly longer travel times than those of nonrural counties: approximately 90 minutes, on average, to the nearest infusion center compared with 45 minutes for nonrural counties, assuming patients would not cross state boundaries.^7^

Other research has correlated travel duration with health care utilization and outcomes to investigate whether longer travel times negatively affect utilization or outcomes. On one hand, some studies have found that longer travel is associated with delayed presentation and diagnosis and increased mortality for several types of cancer.^8,9,10^ On the other hand, longer travel distance has been associated with greater likelihood of receiving surgery and lower operative mortality,^11,12,13^ in some cases despite delays of diagnosis and treatment.^14,15^ Other studies have reported no association between travel times and health care utilization or outcomes.^16,17,18,19^

A possible explanation for this mixed evidence is a lack of normative data for which travel durations are perceived to constitute obstacles to access, how acceptable durations vary by type of medical services, and how perceptions differ by individual characteristics. Without information on what travel times are perceived to be an obstacle to access in different contexts, it is not possible to properly design studies of the association between distance and health care utilization or outcomes. To close this evidence gap, we conducted this study to elicit upper limits for acceptable travel times to primary care, specialty care, and one-time diagnostic procedures from a nationally representative survey panel in the US using a discrete choice experiment.

## Methods

This survey study was approved by the Biomedical Research Alliance of New York Institutional Review Board. Participants provided informed consent electronically. The study followed American Association for Public Opinion Research (AAPOR) best practices on reporting survey research, including details on sample construction, methods used to recruit the sample, survey administration and dates, the sample size and response rate, respondent characteristics, survey mode, the sponsor, the full survey instrument, and associated limitations.^20^

### Data Source

We used the Understanding America Study (UAS), a nationally representative, probability-based internet panel,^21,22,23^ to obtain estimates of participant willingness to travel for routine medical care. A key feature of the UAS is that it samples based on postal addresses to include hard-to-reach populations, who may not have a telephone or internet connection, and provides broadband internet access and a tablet to those who need them. This approach mitigates selection biases facing convenience panels that draw from existing internet users, which harms representativeness, particularly by underrepresenting older adults and individuals with low income.

Our survey was fielded to all UAS panelists aged 65 or older from April 23 to June 8, 2025. The survey captured respondents’ current experiences traveling to medical appointments, including frequency, form of transportation, typical travel times, and problems encountered. Data on self-reported demographic characteristics and health status of the participants were available through regular UAS surveys. Race and ethnicity data were collected to examine differences in willingness to travel along these dimensions. UAS respondents self-report race as American Indian or Alaska Native, Asian, Black or African American, Native Hawaiian or Other Pacific Islander, or White; Hispanic or Latino ethnicity is asked separately. To maintain adequate cell sizes for our statistical analyses, we recoded all races and ethnicities other than non-Hispanic White as racial and ethnic minority. We merged data on self-reported health status from another UAS survey fielded at the same time (May 1-31, 2025).

### Elicitation Method

We conducted a discrete choice experiment that elicited how long participants would be willing to travel to primary care appointments, specialty care appointments, and one-time diagnostic procedures that did not require sedation (eg, a magnetic resonance imaging scan) before they would delay or forgo care. Discrete choice experiments are commonly used to elicit individuals’ preferences in health care settings^24^ on topics ranging from preferences for primary care^25^ to willingness to pay for universal access to Alzheimer disease treatments,^21^ and they have been used to measure willingness to travel in other contexts such as work commutes.^26^ Starting from a randomly chosen travel duration, participants were presented with longer or shorter travel times depending on their prior responses in a range from 5 minutes to 4 or more hours. Details on the procedure and the full survey instrument are contained in eAppendices 1 and 2 and the eFigure in Supplement 1.

We cognitively tested our survey instrument with 8 UAS participants aged 65 or older (not included in the analytic sample) who were purposively selected to have varying educational backgrounds and ages. The interviewees universally and easily understood the survey questions and the trade-offs being presented, as evidenced by their ability to restate questions in their own words and describe their thought process in coming to an answer.

### Analytic Framework

The Behavioral Model of Health Services proposes that health care utilization is predicted by factors that predispose individuals to use services, enable or impede the use of services, and shape their need for services.^27^ We used this framework and the predisposing, enabling, and need factors identified in previous health care utilization studies^28^ to guide the selection of covariates included in the regression analysis. eTable 1 in Supplement 1 describes the selected covariates and their variable construction.

### Statistical Analysis

We described the distributions of respondents’ stated willingness to travel for primary and specialty care appointments and a one-time diagnostic procedure. We used linear regression to explore the associations of sociodemographic characteristics and prior experiences traveling to medical appointments with participant responses regarding willingness to travel. We used 2-sided *t* tests to examine whether regression coefficients were equal to zero, identifying differences as statistically significant if *P* < .05. Analyses were conducted using Stata, version 14.2 (StataCorp LLC).

## Results

### Sample Description

A total of 3390 individuals were invited to participate in the survey. We obtained 2781 responses and restricted the analysis to the 2762 responses without missing covariates. We matched 2650 of our respondents to the survey containing self-reported health status, resulting in a response rate of 78.2% for our analytic sample. Respondents had a mean (SD) age of 72.9 (6.0) years (Table 1); 1377 (52.0%) were female and 1273 (48.0%) were male, 1366 (51.5%) had an annual household income of more than $60 000, and 1218 (46.0%) had a bachelor degree or greater educational attainment. A total of 2081 respondents (78.5%) were non-Hispanic White, and 569 (21.5%) were racial and ethnic minority individuals. Our sample was broadly representative of the US population of older adults, although respondents in this study were more likely to have a bachelor degree or greater (46.0%) than reported by the US Census^29^ (34%). Finally, 506 respondents (19.1%) reported being in fair or poor health, 2039 (76.9%) were retired, and 2071 (78.2%) lived in a metropolitan area.

**Table 1.** Demographic Characteristics of Respondents^a^

Characteristic	Respondents (N = 2650)
Age, mean (SD), y	72.9 (6.0)
Sex	
Female	1377 (52.0)
Male	1273 (48.0)
Annual household income, $	
≤60 000	1284 (48.5)
>60 000	1366 (51.5)
Educational attainment	
Bachelor degree or greater	1218 (46.0)
Less than a bachelor degree	1432 (54.0)
Race and ethnicity	
Non-Hispanic White	2081 (78.5)
Racial and ethnic minority^b^	569 (21.5)
Self-reported health status	
Fair or poor	506 (19.1)
Not fair or poor	2144 (80.9)
Urbanicity	
Lives in a metropolitan area	2071 (78.2)
Lives in a nonmetropolitan area	579 (21.8)
Employment status	
Retired	2039 (76.9)
Not retired	611 (23.1)

^a^ Unless indicated otherwise, values are presented as No. (%) of respondents.

^b^ Understanding America Study respondents self-report race as American Indian or Alaska Native, Asian, Black or African American, Native Hawaiian or Other Pacific Islander, or White; Hispanic or Latino ethnicity is asked separately. To maintain adequate cell sizes for our statistical analyses, we recoded all races and ethnicities other than non-Hispanic White as racial and ethnic minority.

### Prior Experience With Travel for Medical Care

Most respondents (2603 [98.2%]) reported having a primary care physician, and 2275 (85.8%) reported visiting a specialist (Table 2). Among those who visited primary care and specialty care physicians, 2129 (81.8%) indicated that they typically travel 30 minutes or less to a primary care appointment, and 1382 (60.7%) reported doing so for specialty care appointments. We found no differences between respondents living in metropolitan and nonmetropolitan areas in terms of having a primary care physician (2039 [98.5%] vs 564 [97.4%]; *P* = .09) or visiting a specialty care physician (1773 [85.6%] vs 502 [86.7%]; *P* = .51). However, respondents in metropolitan areas were more likely to travel 30 minutes or less to receive primary care (1732 [84.9%] vs 397 [70.4%]; *P* < .001) and specialty care (1237 [69.8%] vs 145 [28.9%]; *P* < .001).

**Table 2.** Travel Experiences of Respondents^a^

Variable	No./total No. of respondents (%)
Has a primary care physician	2603/2650 (98.2)
Travel time to primary care physician (conditional), min	
≤15	1158/2603 (44.5)
16-30	971/2603 (37.3)
31-45	296/2603 (11.4)
46-60	106/2603 (4.1)
>60	72/2603 (2.8)
Visits specialty care physician	2275/2650 (85.8)
Travel time to specialty care physician (conditional), min	
≤15	473/2275 (20.8)
16-30	909/2275 (40.0)
31-45	427/2275 (18.8)
46-60	248/2275 (10.9)
>60	218/2275 (9.6)
Typically drives self to appointments	2222/2650 (83.8)
Is accompanied to medical appointments	961/2650 (36.3)
Reason someone accompanies	
Drive to appointment	369/961 (38.4)
Take notes	280/961 (29.1)
Provide information to physician	136/961 (14.2)
Provide moral or emotional support	520/961 (54.1)
Translate language	17/961 (1.8)
Provide physical assistance	86/961 (8.9)
Other reason	165/961 (17.2)
Has experienced trouble traveling for care	693/2650 (26.2)
Previous problems experienced	
Long travel time	145/693 (20.9)
High trip cost	33/693 (4.8)
Long travel distance	99/693 (14.3)
Unable to find ride	78/693 (11.3)
Disability or limited mobility	118/693 (17.0)
Person to accompany unavailable	85/693 (12.3)
Other problem	173/693 (25.0)

^a^ Respondents were able to select multiple reasons why they were accompanied to medical appointments and multiple reasons why they previously experienced trouble traveling to care.

The majority of respondents (2222 of 2650 [83.8%]) indicated that they most often drive to medical appointments themselves; 961 (36.3%) noted that someone else accompanies them at least occasionally, most commonly to provide moral or emotional support. A total of 693 respondents (26.2%) indicated that they have experienced trouble traveling to medical appointments previously, with long travel times and other troubles being the most common difficulties.

### Willingness to Travel Distributions

The median willingness to travel times were 67.5 (IQR, 37.5-112.5) minutes, 127.5 (IQR, 67.5-232.5) minutes, and 112.5 (IQR, 67.5-202.5) minutes for primary care, specialty care, and one-time diagnostic appointments, respectively (Figure). Notably, most respondents were willing to travel for substantial durations before delaying or forgoing care. Of the 2650 respondents, 1597 (60.3%), 2237 (84.4%), and 2185 (82.5%) were willing to travel at least 1 hour for primary care, specialty care, or one-time diagnostic care, respectively; 260 (9.8%), 562 (21.2%), and 489 (18.5%) respondents stated a willingness to travel 4 hours or more. Median willingness to travel times by covariate are presented in eTable 2 in Supplement 1.

**Figure.**
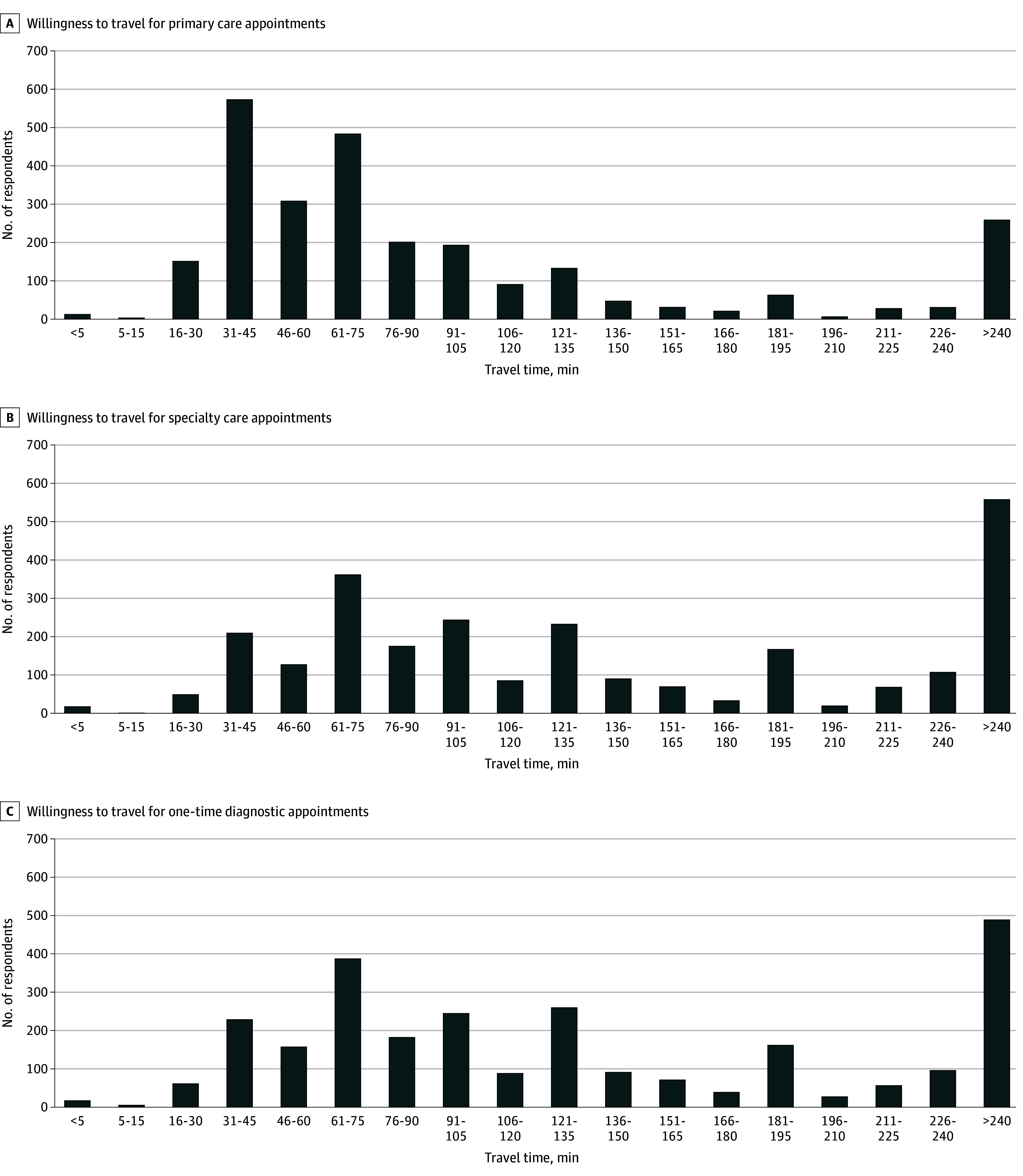
Bar Graphs Depicting Distributions of Willingness to Travel to Primary Care, Specialty Care, and One-Time Diagnostic Appointments Among Older US Adults

### Regression Analysis Results

For predisposing factors, there was no evidence of difference in willingness to travel for routine medical care by sex, but there were significant differences by age, educational attainment, and race and ethnicity. Each year of age was associated with a 0.9-minute reduction (95% CI, −1.7 to −0.2 minutes) in willingness to travel duration for specialty care appointments (*P* = .01); we did not find age-based differences for primary care or diagnostic appointments (Table 3). Individuals with a bachelor degree or greater were willing to travel 17.2 (95% CI, 9.4-25.0) more minutes to a primary care appointment, 25.7 (95% CI, 16.5-35.0) more minutes to a specialty care appointment, and 25.4 (95% CI, 16.5-34.4) more minutes to a one-time diagnostic appointment (all *P* < .001) than those without a bachelor’s degree. Non-Hispanic White respondents were willing to travel 15.3 (95% CI, −24.8 to −5.9) fewer minutes to primary care appointments than racial and ethnic minority individuals (*P* = .002).

**Table 3.** Associations Between Covariates and Respondents’ Willingness to Travel^a^

Variable	Appointment type					
	Primary care (n = 2650)^b^		Specialty care (n = 2649)^c^		One-time diagnostic procedure (n = 2648)^c^	
	Travel time difference, min (95% CI)	*P* value	Travel time difference, min (95% CI)	*P* value	Travel time difference, min (95% CI)	*P* value
Predisposing factor						
Age	−0.03 (−0.7 to 0.6)	.92	−0.9 (−1.7 to −0.2)	.01	−0.4 (−1.1 to 0.3)	.23
Female sex	−2.3 (−9.6 to 5.0)	.54	−5.0 (−13.7 to 3.6)	.25	−0.5 (−9.0 to 8.0)	.91
Bachelor’s degree or greater educational attainment	17.2 (9.4-25.0)	<.001	25.7 (16.5-34.9)	<.001	25.4 (16.5-34.4)	<.001
Non-Hispanic White race	−15.3 (−24.8 to −5.9)	.002	2.4 (−8.4 to 13.2)	.66	−1.6 (−12.1 to 8.8)	.76
Enabling factor						
Annual household income >$60 000	7.4 (−0.5 to 15.2)	.07	22.6 (13.3-31.9)	<.001	19.2 (10.2-28.2)	<.001
Retired	1.6 (−7.1 to 10.3)	.73	4.4 (−6.0 to 14.9)	.40	7.5 (−2.6 to 17.6)	.14
Lives in metropolitan area	−13.1 (−21.9 to −4.2)	.004	−23.9 (−35.2 to −12.7)	<.001	−20.1 (−31.3 to −9.0)	<.001
Typically drives self to care	−1.6 (−12.5 to 9.3)	.77	11.0 (−0.8 to 22.7)	.07	15.8 (4.4-27.2)	.007
Is accompanied to appointments	11.3 (3.0-19.7)	.008	8.4 (−1.3 to 18.0)	.09	12.0 (2.4-21.6)	.01
Trouble traveling in the past	−15.9 (−24.0 to −7.9)	<.001	−20.0 (−30.2 to −9.8)	<.001	−22.1 (−31.8 to −12.4)	<.001
Need factor						
Fair or poor health	−11.0 (−20.0 to −2.1)	.02	−15.0 (−26.1 to −3.9)	.008	−6.8 (−17.7 to 4.1)	.22
*R* ^2^	0.06	NA	0.09	NA	0.08	NA
Dependent variable, mean (SD)	102.9 (95.3)	NA	161.5 (115.3)	NA	152.1 (111.5)	NA

Abbreviation: NA, not applicable.

^a^ Each specification includes a constant and is controlled for the randomized initially presented travel time. Observations vary across specifications due to item nonresponse.

^b^ Controlled for current travel time for primary care using category dummies (including a dummy whether the respondent does not have a primary care physician).

^c^ Controlled for current travel time for specialty care using category dummies (including a dummy for whether the respondent does not visit specialists).

The enabling factors of household income and urbanicity were associated with willingness to travel for medical care, but there were no differences by employment status. Respondents with annual household incomes greater than $60 000 were willing to travel 22.6 (95% CI, 13.3-31.9) more minutes and 19.2 (95% CI, 10.2-28.2) more minutes to specialty care and one-time diagnostic appointments (both *P* < .001), respectively, than those with lower incomes. Individuals who lived in metropolitan areas were willing to travel 13.1 (95% CI, −21.9 to −4.2) fewer minutes to primary care appointments (*P* = .004), 23.9 (95% CI, −35.2 to −12.7) fewer minutes to specialty care appointments (*P* < .001), and 20.1 (95% CI, −31.3 to −9.0) fewer minutes to one-time diagnostic appointments (*P* < .001) than those living in less densely populated areas.

For the other enabling factors, individuals who typically drove themselves to medical appointments were willing to travel 15.8 (95% CI, 4.4-27.2) more minutes (*P* = .007) to receive one-time diagnostic procedures than those who did not. Individuals who were at least occasionally accompanied to their medical appointments were willing to travel 11.3 (95% CI, 3.0-19.7) more minutes to primary care appointments (*P* = .008) and 12.0 (95% CI, 2.4-21.6) more minutes to one-time diagnostic appointments (*P* = .01) than individuals who did not have others accompany them. Individuals who had experienced problems traveling to medical appointments in the past were willing to travel 15.9 (95% CI, −24.0 to −7.9) fewer minutes to primary care appointments, 20.0 (95% CI, −30.2 to −9.8) fewer minutes to specialty care appointments, and 22.1 (95% CI, −31.8 to −12.4) fewer minutes to one-time diagnostic appointments (all *P* < .001) than those who had not experienced trouble traveling to medical appointments.

For the need factor, self-reported health status was selected. Individuals who reported being in fair or poor health were willing to travel 11.0 (95% CI, −20.0 to −2.0) fewer minutes to primary care appointments (*P* = .02) and 15.0 (95% CI, −26.1 to −3.9) fewer minutes to specialty care appointments (*P* = .008) compared with those who self-reported better health.

The findings presented in eTable 3 in Supplement 1 suggest that the randomized starting times had little association with willingness to travel responses, while eTable 4 in Supplement 1 repeats the aforementioned analysis when using an indicator variable capturing whether a respondent reported a willingness to travel 4 or more hours to receive care. We found a similar pattern of results.

## Discussion

To our knowledge, this is the first study to use a nationally representative panel to establish the stated willingness of older US adults to travel for different types of medical care. Our results are in line with a study investigating willingness to travel for ovarian cancer treatment among women presenting at gynecologic oncology clinics in Pennsylvania, which found that 80% of respondents were willing to travel more than 50 miles for care.^30^ Similarly, a survey study of individuals living in the rural Upper Great Plains states suggested that driving distance was not associated with the number of primary and specialty care visits.^31^

Our study challenges assumptions of travel durations that have been hypothesized by researchers to be prohibitive. For instance, the previously mentioned studies on ovarian cancer treatment^30^ and Medicare patients’ travel distance to neurologists^32^ defined long travel as 50 miles or more, which would take about 1 hour in free-flow highway traffic—about half the median time that our respondents were willing to travel for specialty care and one-time diagnostic procedures. Similarly, Li et al^7^ argued that an average driving time of 69 minutes to a one-time diagnostic procedure could constitute a barrier for rural residents, whereas our data would suggest that they are willing to travel at least twice that duration.

Another important finding is that almost all respondents in this nationally representative survey reported having a primary care physician (98.2%) and a specialty physician (85.8%), to whom most (81.8% and 60.7%, respectively) currently traveled 30 minutes or less. These results are similar to those reported in a related study by Ozawa et al,^33^ who conducted a separate survey in the UAS and found that 96% of respondents aged 50 or older reported having a primary care clinician. Similarly, a 2024 Yahoo/YouGov survey^34^ found that 93% of American adults aged 65 or older reported having a primary care physician, while Ganguli et al found that 91% of respondents in the Medicare Current Beneficiary Survey had a usual clinician^35^ and around 80% traveled 30 minutes or less to see them.^36^ Our results are reassuring in light of reports of physician shortages^4,37^ and documented declines in primary care use.^38,39^ However, we cannot rule out, on the basis of our data, that older adults in the US have adjusted to declining capacity by reducing visit frequency or accepting longer wait times for appointments.^38,40^

Individuals in this study with higher incomes and greater educational attainment were willing to travel longer durations to medical appointments. These differences suggest that individuals with higher socioeconomic status can afford higher transportation costs and have more control over their schedules. Ease of access plays a role as well: Respondents who lived in metropolitan areas and those who reported difficulty traveling to medical appointments in the past were willing to travel for shorter durations than their respective counterparts. Conversely, participants who typically drove themselves were willing to travel for longer times for medical appointments than those who relied on other forms of transportation.

Racial and ethnic minority individuals in this study were willing to travel longer times to receive primary care, on average, even after controlling for socioeconomic status and urbanicity of residence. Future research could investigate the underlying causes. Individuals in this study who self-reported being in fair or poor health were also willing to travel shorter durations to receive primary and specialty care. Despite possibly being indicative of having a greater need to receive care, poor health appears to also act as a barrier to willingness to travel for long durations.

Our findings have important policy implications. Older adults’ current level of geographic access and willingness to travel substantial durations for medical care is reassuring in the face of increasing centralization, particularly of specialized care. However, individuals with lower income and lower educational attainment might become vulnerable to geographic barriers to access and may benefit from interventions such as transportation services and availability of telemedicine appointments. Programs such as nonemergency medical transportation benefits offered by Medicaid and some Medicare Advantage plans have been shown to improve access to care,^41,42^ and efforts to increase awareness and usability of these programs may be warranted.^43^

### Limitations

There are several limitations to this study. We elicited stated preferences, which may not align perfectly with actual behavior (revealed preferences). Thus, caution is warranted when interpreting the magnitude of the willingness to travel estimates reported herein, even though our results suggest well-formed preferences. Additionally, our results examining how average willingness to travel times are associated with sociodemographic characteristics varied slightly depending on how we coded travel times for individuals who stated a willingness to travel 4 or more hours one way (the maximum presented travel time), although they remained qualitatively unchanged across sensible assigned values. Differences among racial and ethnic groups should be interpreted with caution because racial and ethnic minority individuals comprised a relatively small portion of our sample (21.5%). Our data cannot be generalized to younger individuals in the US and to other countries. Willingness to travel may also be affected by visit frequency and wait times for appointments, data our study did not collect. Finally, these findings pertain specifically to willingness to travel for routine primary care, specialty care, and one-time diagnostic appointments. Results may differ for other types of medical appointments or procedures, in particular for emergency care.

## Conclusions

The findings of this survey study suggest that older US adults were willing to travel for substantial durations before delaying or forgoing routine medical care. Our findings challenge assumptions regarding travel durations that previous research has assumed to be prohibitive, and they provide important insights into the willingness of older adults to travel to receive routine care in light of increasing health system consolidation. Future research can explore willingness to travel for other types of medical appointments or procedures or for other populations.

## Notes

Supplement 1. **eAppendix 1.** Elicitation Methodology **eFigure.** Willingness to Travel Decision Tree (Starting at 2 Hours) **eTable 1.** Covariates Included in the Regression Analysis **eTable 2.** Median Travel Times by Appointment Type and Respondent Characteristics **eTable 3.** Stated Willingness to Travel: Influence of Randomized Start Times **eTable 4.** Associations Between Covariates and Stated Willingness to Travel 4 or More Hours **eAppendix 2.** Survey Instrument

Supplement 2.
Data Sharing Statement
