# Supplementary material for: Willingness of Older Adults to Travel for Medical Care
Source: JAMA Netw Open. 2026 Feb 23;9(2):e2560280. doi: 10.1001/jamanetworkopen.2025.60280 (PMC12931471; doi:10.1001/jamanetworkopen.2025.60280)
Supplement: Supplement 1. — eAppendix 1. Elicitation Methodology eFigure. Willingness to Travel Decision Tree (Starting at 2 Hours) eTable 1. Covariates Included in the Regression Analysis eTable 2. Median Travel Times by Appointment Type and Respondent Characteristics eTable 3. Stated Willingness to Travel: Influence of Randomized Start Times eTable 4. Associations Between Covariates and Stated Willingness to Travel 4 or More Hours eAppendix 2. Survey Instrument [file jamanetwopen-e2560280-s001.pdf]

Supplemental Online Content

Burke J, Ozawa T, Liu Y, Ye W, Mattke S. Willingness of older adults to travel for medical care. *JAMA Netw Open*. 2026;9(2):e2560280. doi:10.1001/jamanetworkopen.2025.60280

eAppendix 1. Elicitation Methodology

eFigure. Willingness to Travel Decision Tree (Starting at 2 Hours)

eTable 1. Covariates Included in the Regression Analysis

eTable 2. Median Travel Times by Appointment Type and Respondent Characteristics

eTable 3. Stated Willingness to Travel: Influence of Randomized Start Times

eTable 4. Associations Between Covariates and Stated Willingness to Travel 4 or More Hours

eAppendix 2. Survey Instrument

This supplemental material has been provided by the authors to give readers additional information about their work.

© 2026 Burke J et al. *JAMA Network Open*.

eAppendix 1. Elicitation Methodology

To elicit willingness to travel, we presented respondents with scenarios of the following form for each of the types of care we investigate:

*"Before you delay or forgo care, would you travel **one hour** one-way to a primary care appointment?*

- *Yes, I would travel **one hour** one-way*
- *No, I would delay or forgo care"*

To examine possible anchoring effects and stability of preferences, we randomized the initial bolded travel duration in each scenario for each participant to be either one hour, two hours, or three hours. Subsequent questions presented either shorter or longer travel durations depending on the participant's prior response. The sequence of questions was structured to either (1) bound participants' willingness to travel in a 15-minute window, (2) determine they were willing to travel 4 or more hours one-way to the appointment, or (3) determine they were willing to travel fewer than 5 minutes one-way to the appointment. For responses bounded by a 15-minute window (e.g. between 15 and 30 minutes), we code willingness to travel at the midpoint. For respondents who indicated they'd be willing to travel 4 or more hours one-way, we code willingness to travel to be 1.5 times the maximum travel time presented (i.e. to be 6 hours).[1] For those willing to travel less than 5 minutes, we set willingness to travel equal to ½ the minimum travel time presented (i.e. to be 2.5 minutes). The order with which the primary care, specialty care, and one-time diagnostic procedures scenarios were presented was randomized across participants. Figure A1 presents the decision tree when the initial presented travel time was 2 hours.

[1] Our results are qualitatively unchanged with other codings such as 1x or 2x the maximum presented travel length.

© 2026 Burke J et al. *JAMA Network Open*.

eFigure. Willingness to Travel Decision Tree (Starting at 2 Hours)

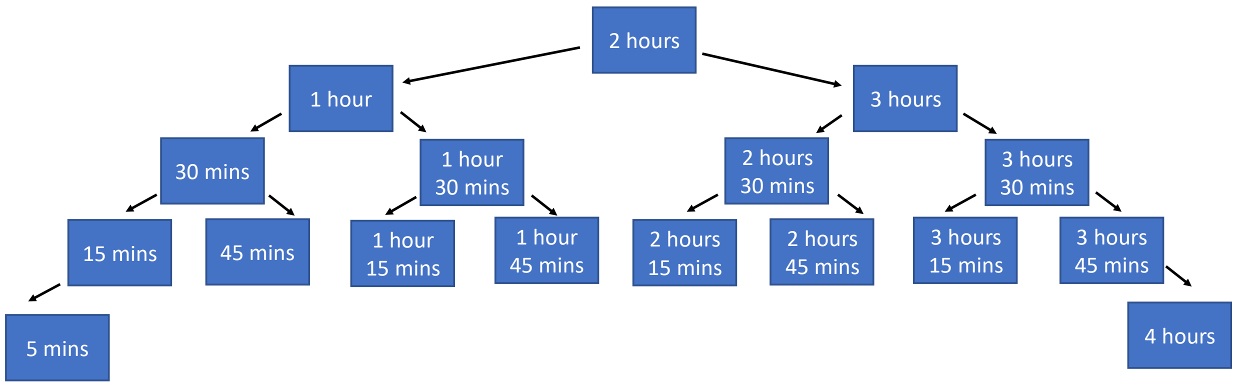

Across each appointment type, we randomized whether respondents were first presented with a decision to travel one hour, two hours, or three hours to receive care. Importantly, we find little effect from our randomized starting travel durations on responses (Appendix Table A2). None of the randomized start times had statistically significant effects at conventional levels on reported willingness to travel times, outside of a difference of approximately 11 minutes (p-value = 0.02), on average, between those starting with two- hour travel times and those starting with three-hour travel times for trips for primary care appointments. This indicates that our respondents had well-formed preferences, were not unduly swayed by randomly selected starting values, and gives confidence that respondents took the task seriously and provided thoughtful answers.

© 2026 Burke J et al. *JAMA Network Open*.

Covariates included in the Regression Analyses

The Behavioral Model of Health Services proposes that healthcare utilization is predicted by factors that predispose individuals to use services, enable or impede the use of services, and shape their need for services. We used this framework to guide the selection of covariates included in the regression analysis. The predisposing factors we selected were age, sex, education (greater or less than bachelor's degree), and race (non-Hispanic white or other race/ethnicity). The enabling factors were household income (greater or less than $60,000 per year), employment status (retired or not retired), self-reported urbanicity, and prior experiences traveling to medical care, including whether the individual typically drove themselves to care, whether they were at least occasionally accompanied to appointments, and whether they reported having trouble traveling to medical care in the past. Finally, we selected self-reported health status as the need factor. Appendix Table A1 describes how each of the covariates were constructed.

© 2026 Burke J et al. *JAMA Network Open*.

eTable 1. Covariates Included in the Regression Analysis

Covariate	Description
<u>Predisposing Factors</u>	
**Age**	Respondent's age in years
Female	= 1 if respondent reports sex as female
Bachelors or more education	= 1 if respondent reports having a bachelor's degree, master's degree, professional school degree, or doctorate degree
Non-Hispanic White	= 1 if respondent reports race as White and does not report being Hispanic or Latino
<u>Enabling Factors</u>	
Annual Income > $60k	= 1 if respondent reports that annual household income exceeds $60,000
Retired	= 1 if respondent reports being retired
**Lives in metropolitan area**	= 1 if respondent lives in a census tract classified as metropolitan
**Typically drives self to care**	= 1 if respondent reports most often traveling to medical appointments by driving themselves
**Accompanied to appointments**	= 1 if respondent reports being accompanied to medical appointments most of the time, sometimes, or occasionally
**Trouble traveling in the past**	= 1 if respondent does not report never experiencing trouble traveling to medical appointments
<u>Need Factor</u>	
Fair or poor health	= 1 if respondent reports being in fair health or poor health

Notes: UAS respondents self-report race as American Indian or Alaska Native, Asian, Black or African American, Native Hawaiian or Other Pacific Islander, or White. Hispanic/Latino ethnicity is asked separately. To maintain adequate cell sizes for our statistical analyses, we recoded all races and ethnicities other than non-Hispanic white as "other."

© 2026 Burke J et al. *JAMA Network Open*.

eTable 2. Median Travel Times by Appointment Type and Respondent Characteristics

VARIABLES	(1)<br>Primary Care Appointment	(2)<br>Specialty Care Appointment	(3)<br>Diagnostic Appointment
Predisposing Factors			
Age > 75	67.5 (37.5, 112.5)	127.5 (67.5, 217.5)	127.5 (67.5, 187.5)
Female	67.5 (37.5, 97.5)	112.5 (67.5, 217.5)	97.5 (67.5, 187.5)
Bachelors or more education	67.5 (52.5, 127.5)	127.5 (82.5, 360)	127.5 (67.5, 232.5)
Non-Hispanic White	67.5 (37.5, 112.5)	127.5 (67.5, 232.5)	127.5 (67.5, 202.5)
Enabling Factors			
Annual income > $60K	67.5 (37.5, 127.5)	127.5 (82.5, 360)	127.5 (67.5, 232.5)
Retired	67.5 (37.5, 112.5)	127.5 (67.5, 232.5)	112.5 (67.5, 202.5)
Lives in metropolitan area	67.5 (37.5, 112.5)	112.5 (67.5, 217.5)	97.5 (67.5, 187.5)
Typically drives self to care	67.5 (37.5, 112.5)	127.5 (67.5, 232.5)	112.5 (67.5, 217.5)
Accompanied to appointments	67.5 (37.5, 127.5)	127.5 (67.5, 232.5)	127.5 (67.5, 217.5)
Trouble traveling in past	67.5 (37.5, 112.5)	112.5 (67.5, 187.5)	97.5 (67.5, 187.5)
Need Factors			
Fair or Poor Health	67.5 (37.5, 97.5)	112.5 (67.5, 187.5)	112.5 (67.5, 187.5)
Observations	2,650	2,649	2,648
Sample Median	67.5 (37.5, 112.5)	127.5 (67.5, 232.5)	112.5 (67.5, 202.5)

Notes: The table presents median travel times by respondent characteristics. 25th and 75th percentiles are in parentheses. Age > 75 = 1 for respondents above age 75 and the rest of the covariates are as defined in Table A1.

© 2026 Burke J et al. *JAMA Network Open*.

eTable 3. Stated Willingness to Travel: Influence of Randomized Start Times

VARIABLES	(1)<br>Primary Care Appointment	(2)<br>Specialty Care Appointment	(3)<br>Diagnostic Appointment
Start at 1 hour	-0.98 (-9.31, 7.34)	-5.91 (-16.17, 4.35)	0.47 (-9.52, 10.46)
Start at 3 hours	11.07** (2.18, 19.97)	3.19 (-7.12, 13.50)	6.99 (-2.97, 16.94)
Observations	2,650	2,649	2,648
R-squared	0.06	0.09	0.08
Mean Dependent Variable	102.9	161.5	152.1

Notes: This table presents results from the same specifications estimated in Table 3 and shows the impacts of the assigned randomized start times. 95% confidence intervals are in parentheses. Column (1) controls for current drive time for primary care using category dummies (including a dummy whether the respondent doesn't have a primary care doctor), while columns (2) - (3) control for current drive time for specialty care using category dummies (including a dummy for whether the respondent doesn't visit specialists). Observations vary across specifications due to item non-response. Each specification controls for covariates in Table 1 and Table 2. * $p < 0.10$, ** $p < 0.05$, *** $p < 0.01$.

© 2026 Burke J et al. *JAMA Network Open*.

eTable 4. Associations Between Covariates and Stated Willingness to Travel 4 or More Hours

VARIABLES	(1)<br>Primary Care Appointment	(2)<br>Specialty Care Appointment	(3)<br>Diagnostic Appointment
Predisposing Factors			
Age	-0.00 (-0.00, 0.00)	-0.00*** (-0.01, -0.00)	-0.00* (-0.01, -0.00)
Female	0.01 (-0.02, 0.03)	-0.01 (-0.04, 0.03)	0.00 (-0.03, 0.03)
Bachelors or more education	0.03** (0.01, 0.06)	0.06*** (0.03, 0.10)	0.07*** (0.04, 0.10)
Non-Hispanic White	-0.04*** (-0.07, -0.01)	-0.01 (-0.05, 0.02)	-0.03 (-0.06, 0.01)
Enabling Factors			
Annual income > $60K	0.02* (-0.00, 0.05)	0.07*** (0.03, 0.10)	0.06*** (0.03, 0.09)
Retired	0.01 (-0.02, 0.04)	0.02 (-0.02, 0.06)	0.02 (-0.02, 0.05)
Lives in metropolitan area	-0.01 (-0.04, 0.01)	-0.05** (-0.09, -0.01)	-0.03 (-0.07, 0.01)
Typically drives self to care	-0.02 (-0.05, 0.02)	0.03 (-0.01, 0.07)	0.04** (0.00, 0.08)
Accompanied to appointments	0.02* (-0.00, 0.05)	0.02 (-0.02, 0.05)	0.04** (0.01, 0.08)
Trouble traveling in past	-0.05*** (-0.07, -0.02)	-0.05*** (-0.09, -0.01)	-0.07*** (-0.10, -0.03)
Need Factors			
Fair or Poor Health	-0.02* (-0.05, 0.01)	-0.05**(-0.09, -0.01)	-0.02 (-0.06, 0.02)
Observations	2,650	2,649	2,648
R-squared	0.02	0.04	0.04
Mean Dependent Variable	0.10	0.21	0.18

Notes: 95% confidence intervals are in parentheses. Each specification includes a constant and controls for the randomized initial presented travel time. Column (1) controls for current drive time for primary care using category dummies (including a dummy whether the respondent doesn't have a primary care doctor), while columns (2) - (3) control for current drive time for specialty care using category dummies (including a dummy for whether the respondent doesn't visit specialists). Observations vary across specifications due to item non-response. * p < 0.10, ** p < 0.05, *** p < 0.01.

© 2026 Burke J et al. *JAMA Network Open*.

eAppendix 2. Survey Instrument

We would like to ask you about traveling to medical appointments, such as doctor's visits, trips to testing facilities, or trips for medical procedures.

Q1. In the past 12 months, how frequently did you travel to medical appointments?
- 1-4 times
- 5-8 times
- 9-12 times
- More than 12 times
- I did not travel to medical appointments in the past 12 months

Q2. How do you most often travel to medical appointments?
- By driving myself
- By taxi or ride-hailing service (e.g., Uber, Lyft, etc.)
- By private vehicle as a passenger (car, van, carpool, truck)
- By public transportation
- By other means

Q3. How long do you typically travel to see your **primary care doctor**?
- 0-15 minutes
- 16-30 minutes
- 31-45 minutes
- 46-60 minutes
- 61-90 minutes
- More than 90 minutes
- I don't have a primary care doctor

Q4. How long do you typically travel to see **a specialist doctor**, such as a cardiologist or neurologist?
- 0-15 minutes
- 16-30 minutes
- 31-45 minutes
- 46-60 minutes
- 61-90 minutes
- More than 90 minutes
- I don't see specialty care doctors

Q5. Does someone accompany you to medical appointments?
- Yes, most of the time
- Yes, sometimes
- Yes, but only occasionally
- No [--->skip to Q8]

© 2026 Burke J et al. *JAMA Network Open*.

[If Q5=any of the “yes” options]

Q6. What are the reasons why someone accompanies you to medical appointments? Please check all that apply. [randomize the order of the options except for “Other”]

- Drive me to the appointment
- Take notes or remember what the doctor says
- Explain my condition or give information to the doctor
- Provide moral or emotional support
- Translate language
- Provide physical assistance (e.g., help up or down stairs)
- Other, please specify [add a blank text box]

Q7. Among the reasons you selected above, which is the ***most important reason*** why someone accompanies you to medical appointments?

[populate the options R selects from the question above]

Q8. How often do you experience problems travelling to medical appointments?

- Never
- Rarely
- Sometimes
- Most of the time
- Always

[If Q8 is NOT “never”]

Q9. What are the reasons why you experience problems travelling to medical appointments? Please check all that apply.

- Long travel time
- High trip cost
- Long travel distance
- Unable to find a ride
- Disability or limited mobility
- Person to accompany me unavailable
- Other, please specify [add a blank text box]

Q10. Among the reasons you selected above, which is the **most important reason** why you experience problems travelling to medical appointments?

[populate the options R selects from the question above]

© 2026 Burke J et al. *JAMA Network Open*.

Now, we would like to know how long you would be willing to travel one-way to a **{ primary care/ specialty care (such as neurologist or cardiologist) /one-time diagnostic procedure (such as an MRI)}** appointment **<u>before you would delay or forgo care</u>**.

Before you delay or forgo care, would you travel **{X duration}** one-way to a **{primary care/specialty care/one-time diagnostic procedure}** appointment?

- Yes, I would travel **{X duration}** one-way
- No, I would delay or forgo care

© 2026 Burke J et al. *JAMA Network Open*.
