# Supplementary material for: Willingness of Older Adults to Travel for Medical Care
Source: JAMA Netw Open. 2026 Feb 23;9(2):e2560280. doi: 10.1001/jamanetworkopen.2025.60280 (PMC12931471; doi:10.1001/jamanetworkopen.2025.60280)
Supplement: Supplement 2. — Data Sharing Statement [file jamanetwopen-e2560280-s002.pdf]

Data Sharing Statement

Burke. Willingness of Older Adults to Travel for Medical Care. *JAMA Netw Open*. Published February 23, 2026. doi:10.1001/jamanetworkopen.2025.60280

Data
Data available: Yes
Data types: Deidentified participant data, Data dictionary
How to access data: The raw survey data will be available on the UAS website upon paper publication.
When available: With publication

Supporting Documents
Document types: Other (please specify)
Additional Information: survey text
How to access documents: Part of the published supplement
When available: With publication

Additional Information
Who can access the data: under DUA
Types of analyses: any purpose
Mechanisms of data availability: under DUA, without investigator support
Any additional restrictions: N/A
